# Supplementary material for: Cross-regional real-time visualization of systemic physiology and dynamics with 3D panoramic photoacoustic computed tomography (3D-PanoPACT)
Source: Nat Commun. 2025 Nov 18;16:10077. doi: 10.1038/s41467-025-65054-x (PMC12627095; doi:10.1038/s41467-025-65054-x)
Supplement: Supplementary file 1 — Supplementary Information [file 41467_2025_65054_MOESM1_ESM.pdf]

Supplementary Information

Cross-regional real-time visualization of systemic physiology and dynamics with 3D panoramic photoacoustic computed tomography (3D-PanoPACT)

Supplementary Figures

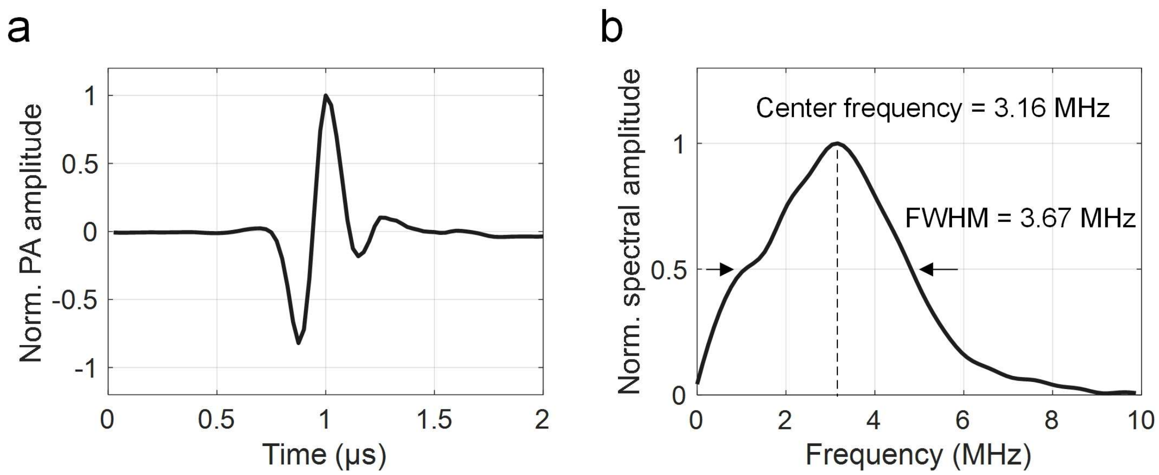

Figure S1. Characterization of system bandwidth.

a The waveform of impulse response in 3D-PanoPACT. **b** The Fourier transform of impulse response characterizing the system bandwidth. FWHM, full width at half maximum.

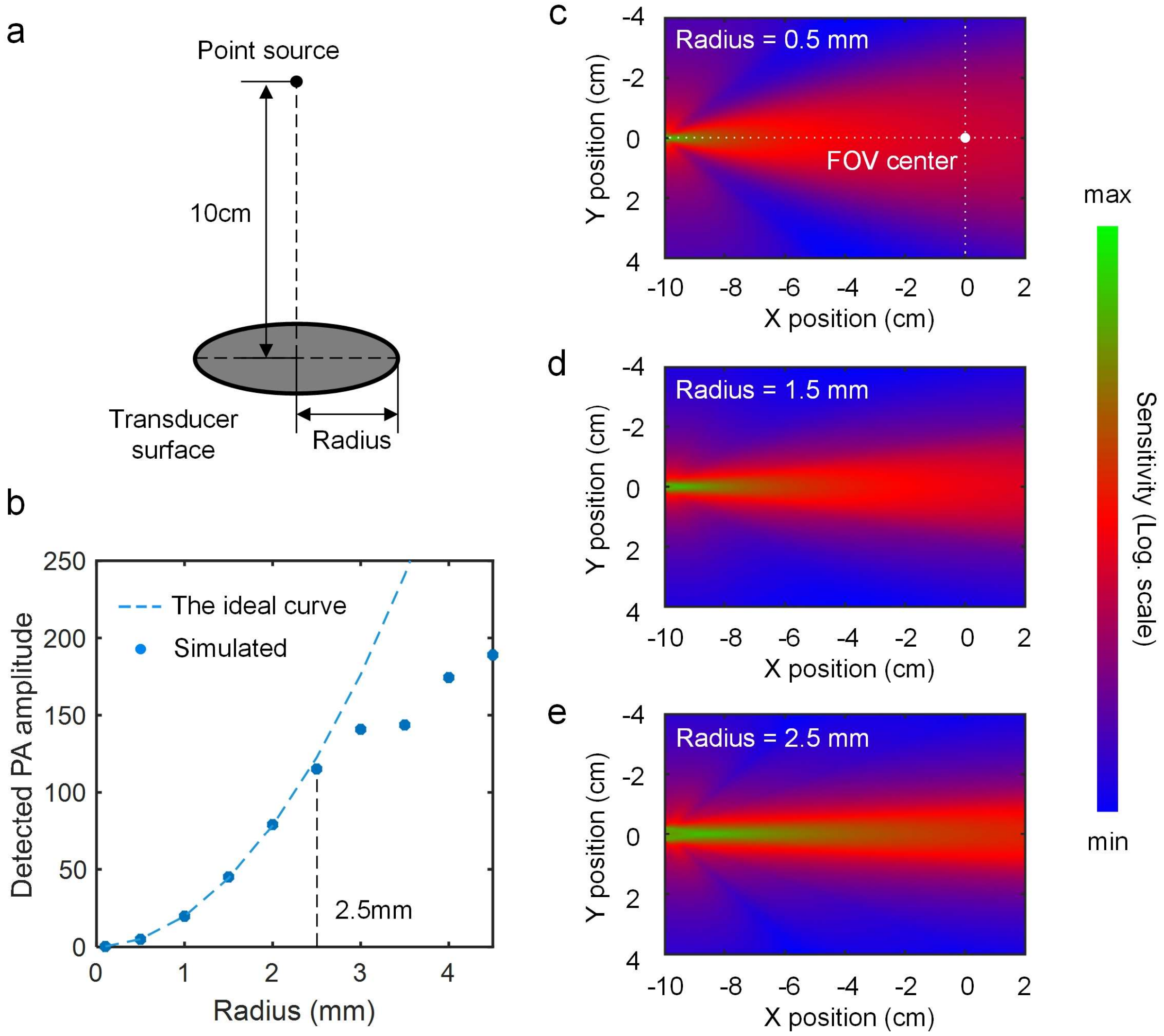

Figure S2. Design of transducer element size.

a The simulation setup. **b** Simulation results of the relation between element radius and detected PA amplitude. **c-e** Simulation of transducer element sensitivity distribution with radius of 0.5 mm, 1.5 mm and 2.5 mm, respectively.

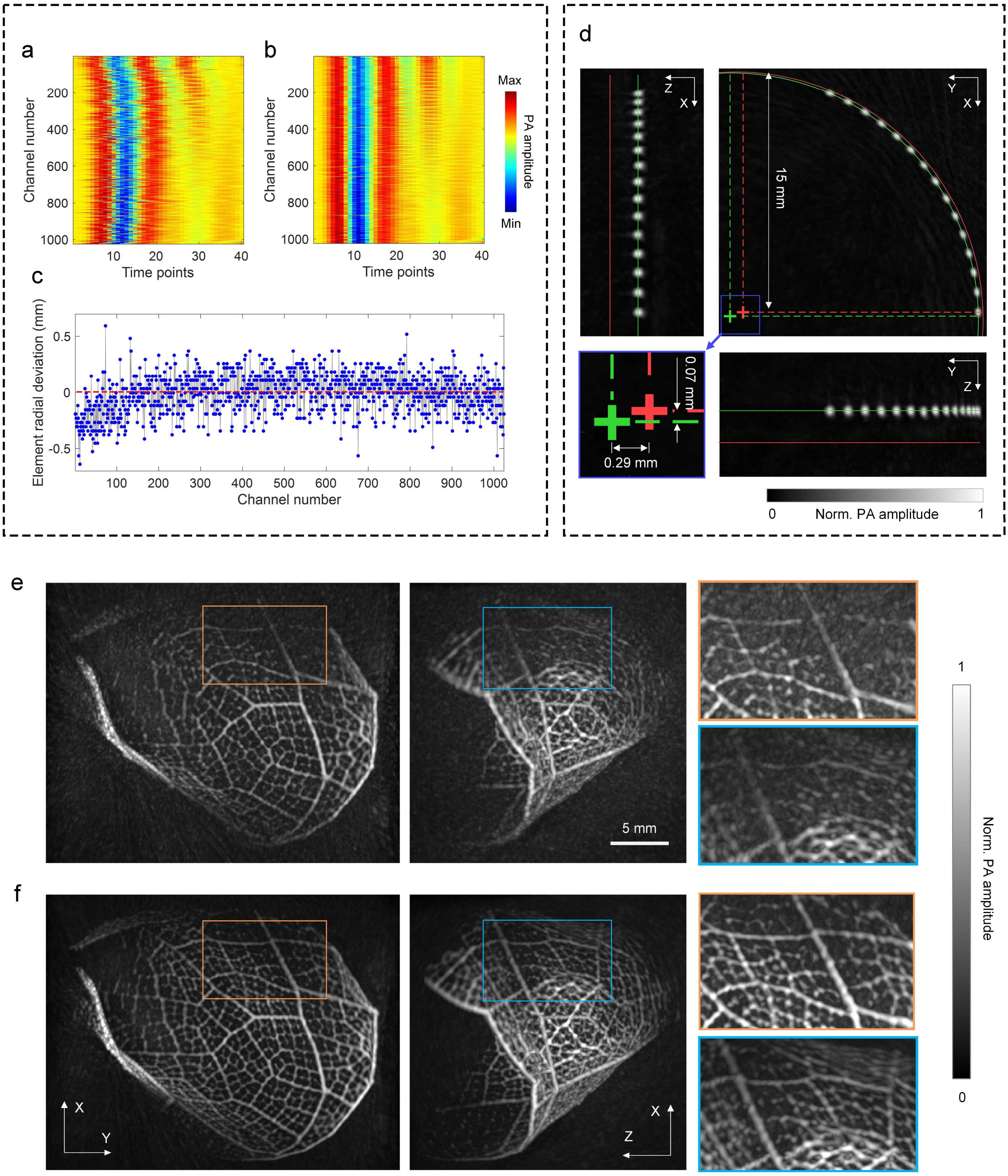

Figure S3. Geometric calibrations in 3D-PanoPACT.

a-c Calibrations of the element position. **d** Calibrations of the system rotation axis. **a** The raw data from a point absorber without position correction. **b** The corrected raw data from a point absorber after calibration. **c** The radial deviation of the 1024 transducer elements. **d** The reconstructed image of the chain of absorbers is shown in different views. The ideal rotation axis and tracks are plotted in red, while the actual ones are

in green. Through the calculation of analytical geometry, the actual axis is parallel to the ideal axis, which is only shifted 0.07 mm along the X-axis and 0.29 mm along the Y-axis, as labelled. **e** The reconstructed image of the leaf skeleton without calibrations. **f** The reconstructed image after the geometric calibrations. Two representative parts are zoomed in to observe the improvements.

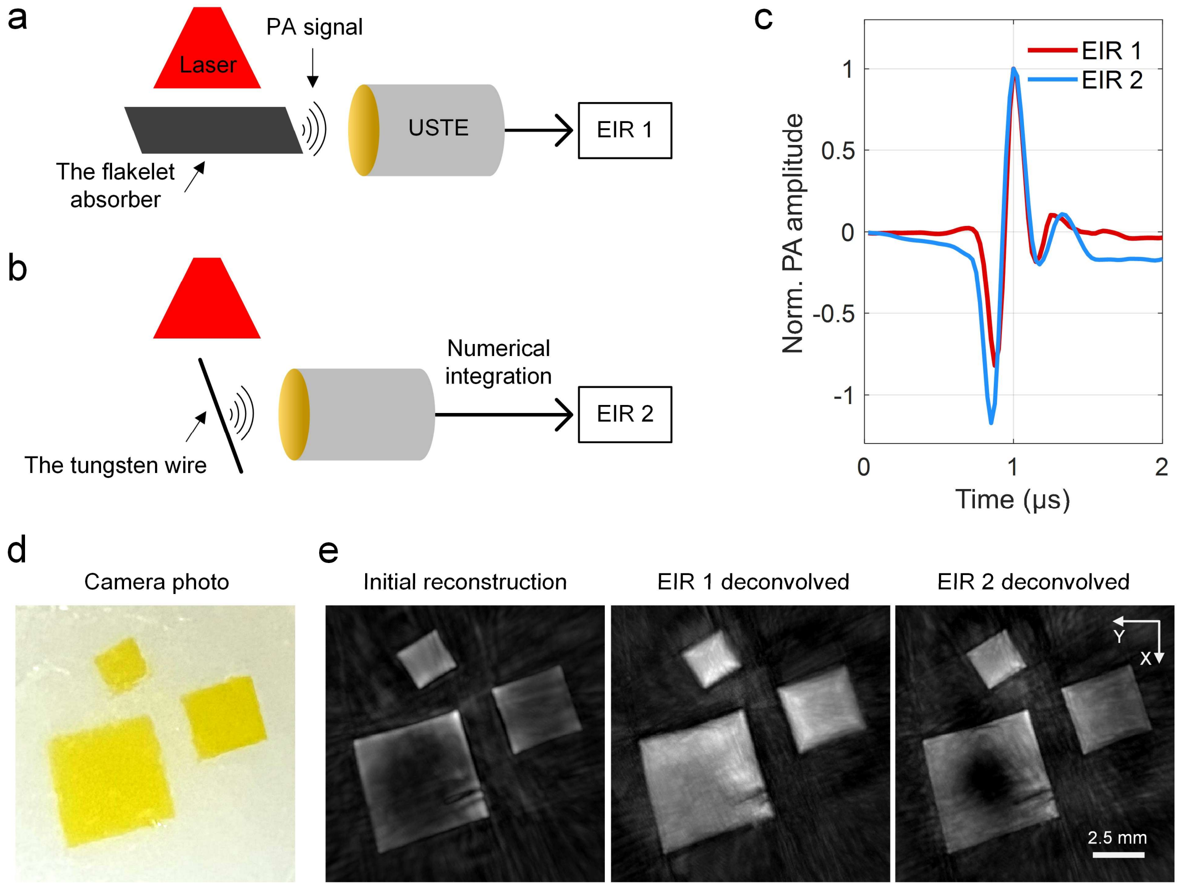

Figure S4. The calibration of EIR and image deconvolution.

a The test method of EIR 1 using a flakelet absorber. **b** The test method of EIR 2 using a tungsten wire and numerical integration. **c** The waveforms of EIR 1 and EIR 2. **d** The camera photo of the phantom. **e** The reconstructed images of a phantom with low-frequency features using three different processing methods, separately.

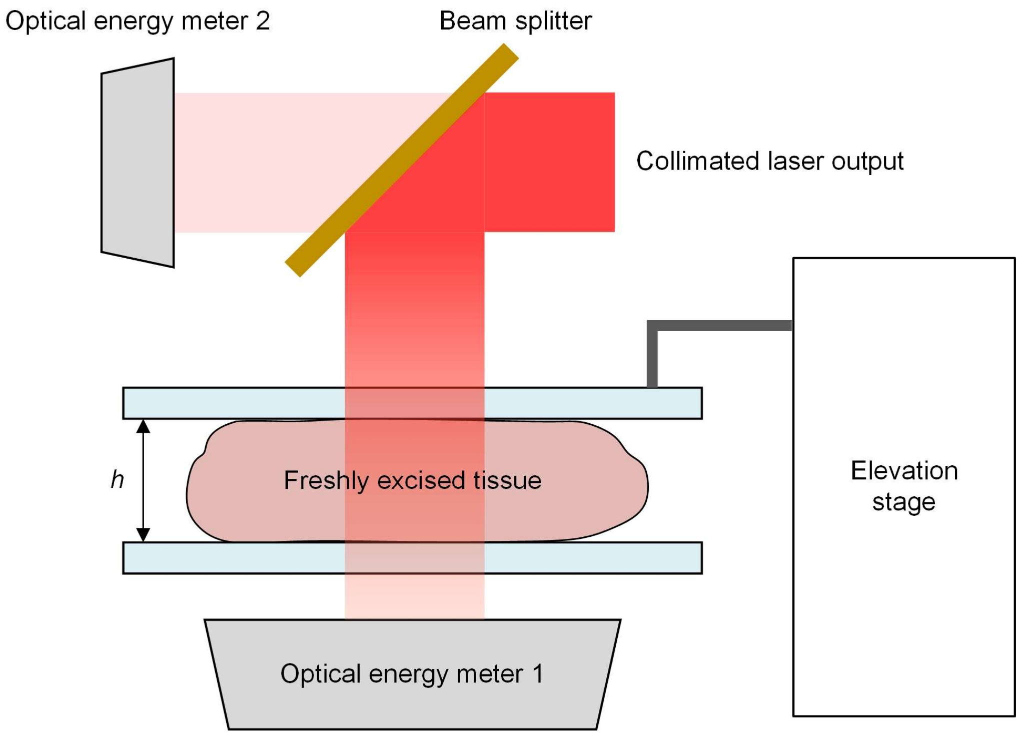

Figure S5. The setup for optical attenuation coefficient estimation in fresh tissue.

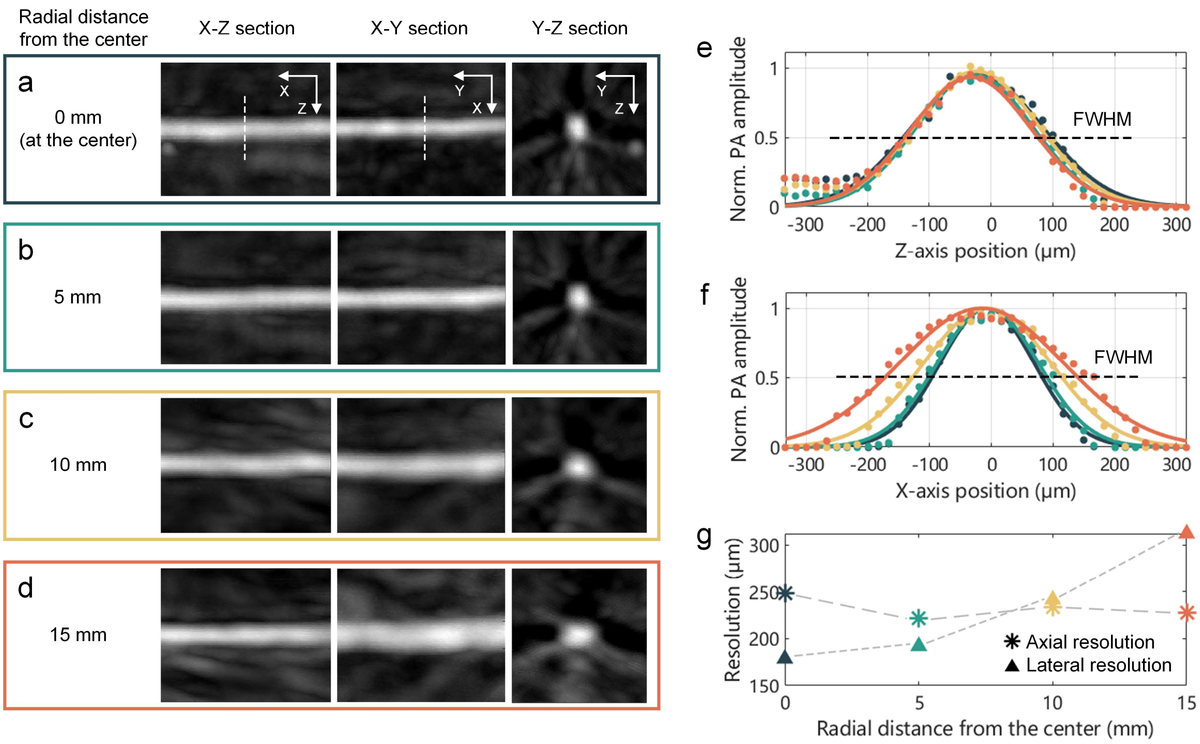

Figure S6. Characterization of spatial resolution in 3D-PanoPACT.

a-d The reconstructed images of a 10-µm-diameter tungsten wire at a radial distance of 0 mm, 5 mm, 10 mm, and 15 mm from the center within the FOV, respectively. Three orthogonal sections are shown. **e** The Gaussian-fitted profile in X-Z sections (dashed line in **a**) from different radial positions showing the axial resolution (characterized by FWHM value). The color of the curves corresponds to the rectangular boxes and is used to represent the radial position. **f** The Gaussian-fitted profile in X-Y sections (dashed line in **a**) from different radial positions showing the lateral resolution. **g** The spatial resolution changes with the radial position at 0 mm, 5 mm, 10 mm, and 15 mm. Axial resolution: 248.1 µm, 221.5 µm, 233.2 µm, 227.5 µm. Lateral resolution: 178.6 µm, 192.1 µm, 241.7 µm, 312.3 µm.

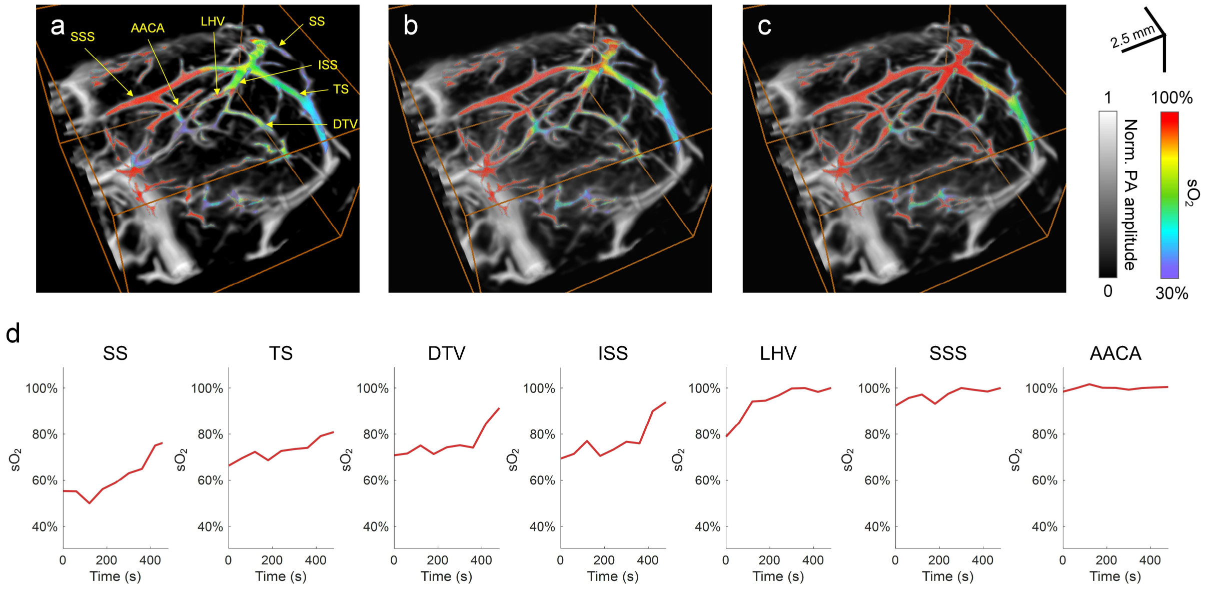

Figure S7. Real-time monitoring of the whole-brain hemodynamics in the recovery process after the discontinuation of anesthesia.

a-c The whole-brain sO_2 maps superimposed on the anatomy background acquired at 10 s, 240 s and 480 s after the discontinuation of anesthesia, respectively. The imaging rate is 0.5 Hz. **d** The changing curve of sO_2 over 490 s in different vessels labelled in **a**. SS, sigmoid sinus; TS, transverse sinus; DTV, dorsal thalamic vein; ISS, inferior sagittal sinus; LHV, longitudinal hippocampal vein; SSS, superior sagittal sinus; AACA, azygos of the anterior cerebral artery

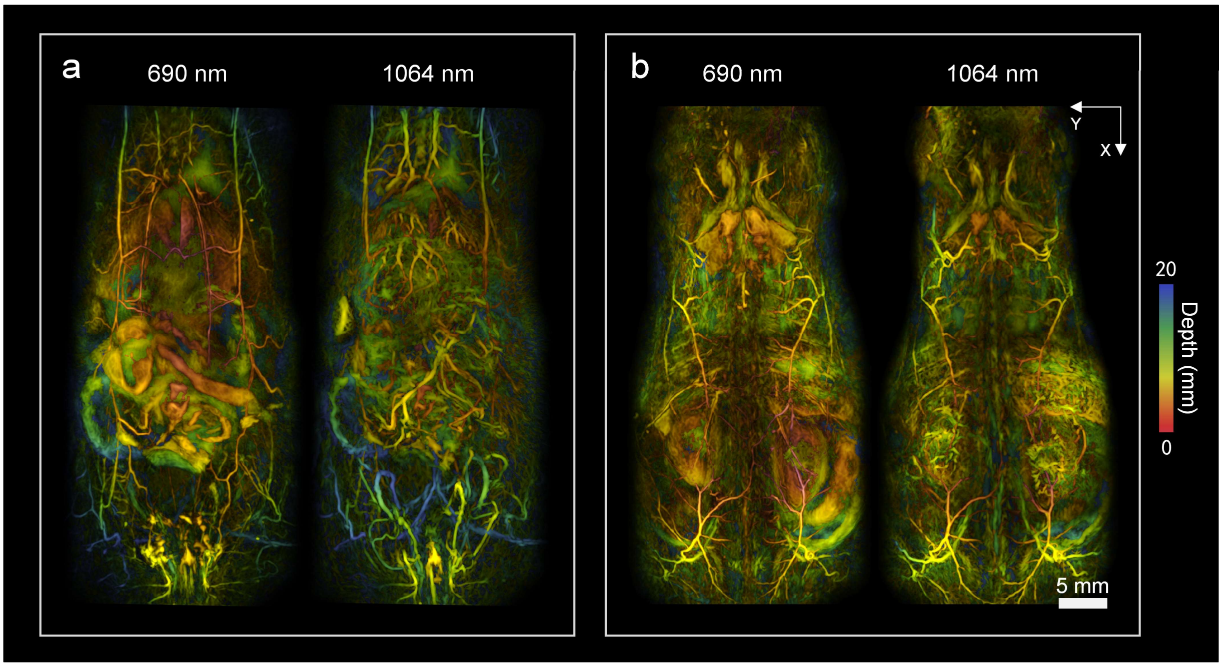

Figure S8. The comparison of features of the whole trunk in dual-wavelength imaging.
a The frontal view of the whole trunk imaged at 690 nm and 1064 nm. Remarkable feature differences are observed. The image at 690 nm presents the superficial vessels and the digestive system, while the image at 1064 nm reveals the sub-organ vascular networks. **b** The backside view of the whole trunk imaged at 690 nm and 1064 nm.

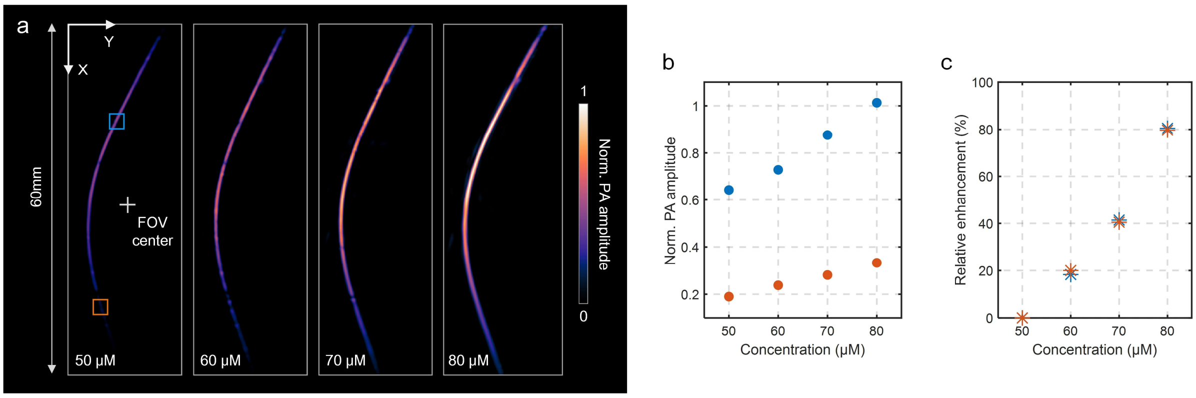

Figure S9. Validation of A1094 probe concentration tracking using relative change.

a Imaging results of serial dilutions of A1094 in the tube. **b** Image pixel values acquired from regions of interest (colored boxes in **a**) in different concentrations. Data markers are color-coded to correspond with their respective regions. **c** Relative signal percentage change with 50 μM as reference baseline.

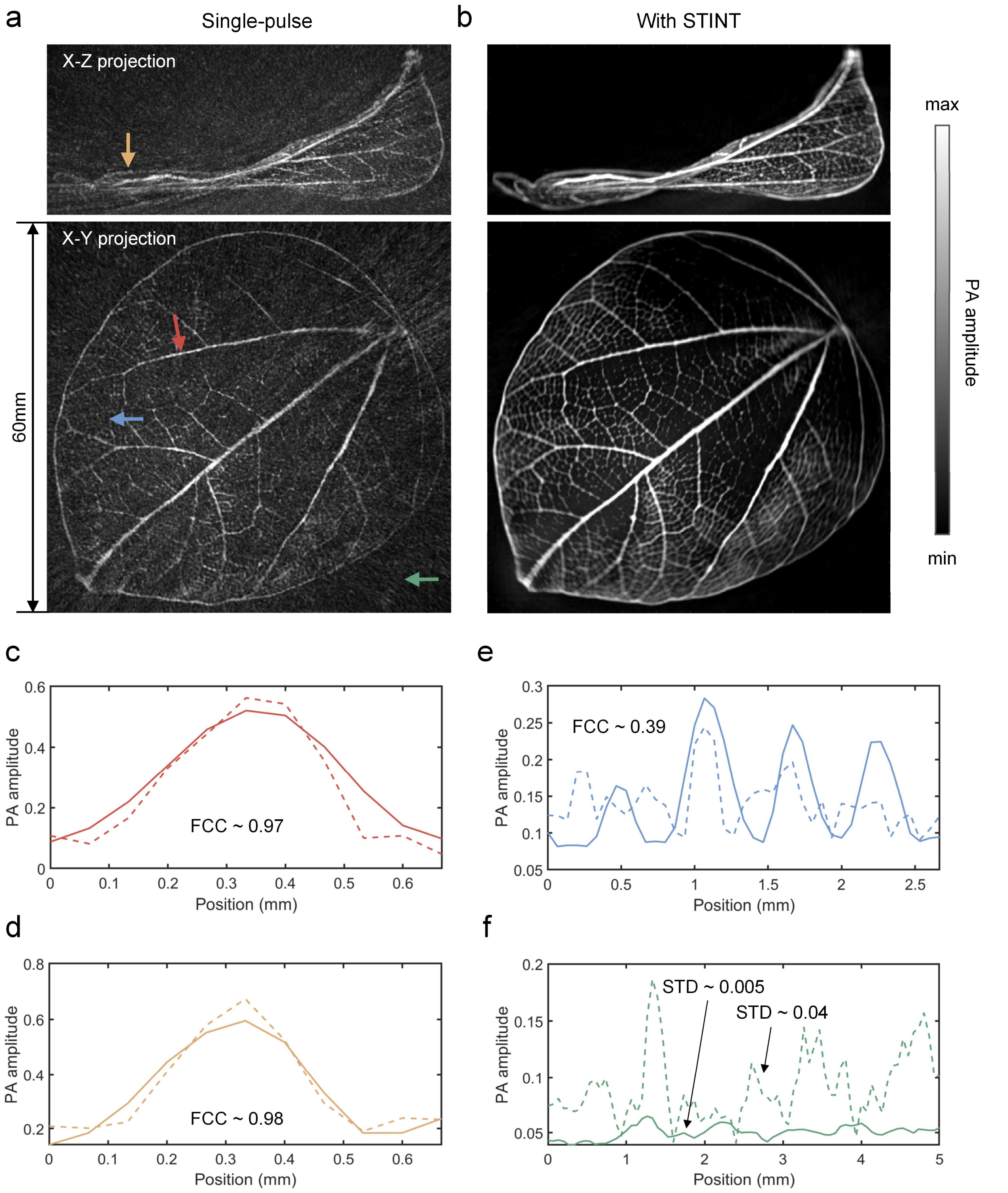

Figure S10. Quantification of the STINT method's effectiveness.

a The single-pulse 3D image results of a complete leaf vein. **b** The results with the STINT method. **c-f** Profile comparison, where the single-pulse image is represented by dashed lines, and the results obtained using the STINT method are indicated by solid lines. Colors correspond to the arrow positions in **a**. FCC, feature correlation coefficient. STD, the standard deviation.

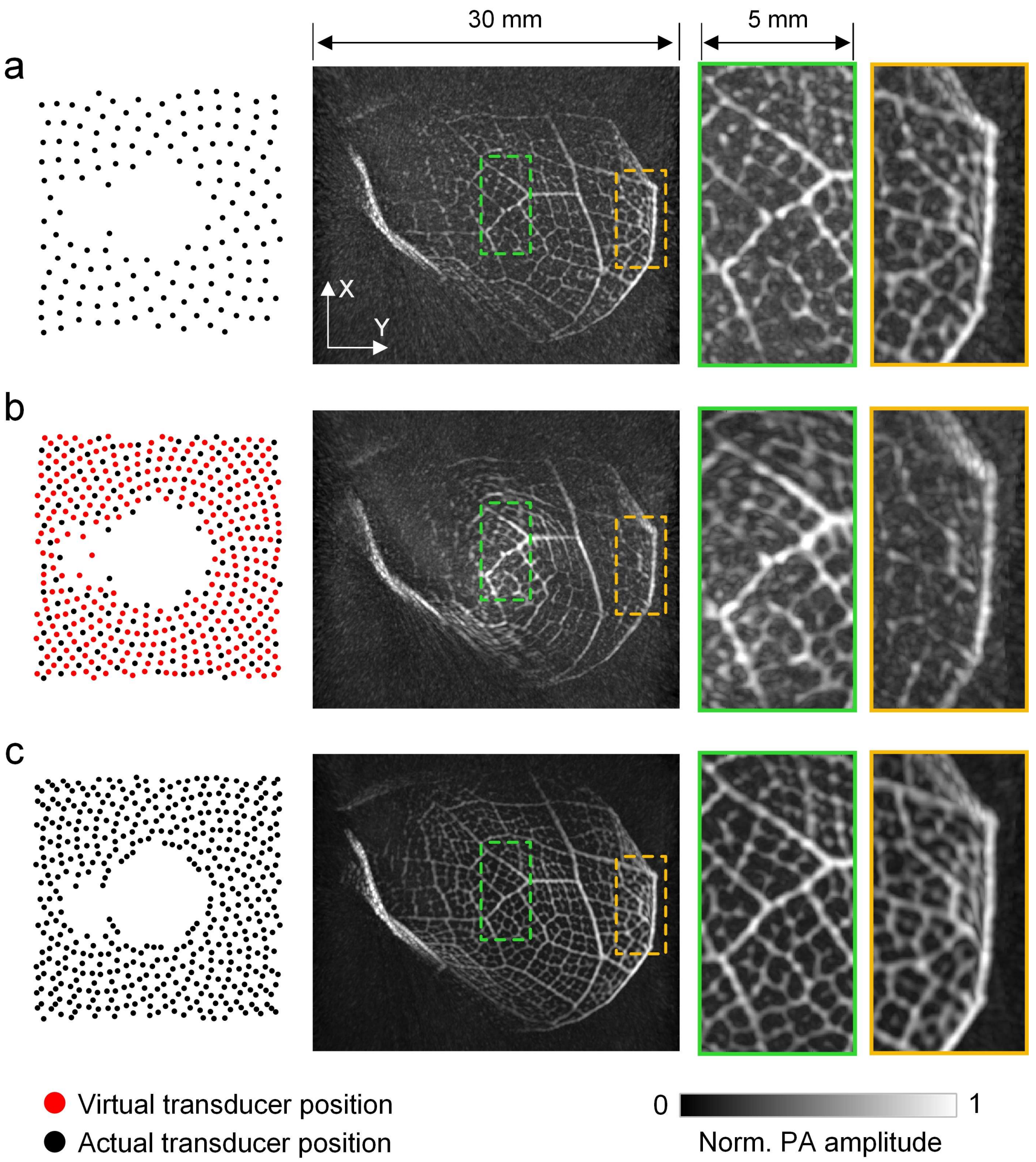

Figure S11. The comparison of STINT method and numerical spatial interpolation.

a The schematic diagram of the original 1024-element array (top view) and the reconstructed images. Two zoomed-in regions are displayed for a more detailed comparison (the same below). **b** The schematic diagram of the numerical-spatial-interpolated array and the reconstructed images using the interpolated raw data. The actual transducers are shown in black while the virtual (interpolated) ones are in red. **c** The schematic diagram of the equivalent array using the STINT method and the corresponding reconstructed images. For effective comparison, the total number of transducers is consistent with the interpolated results in **b**.

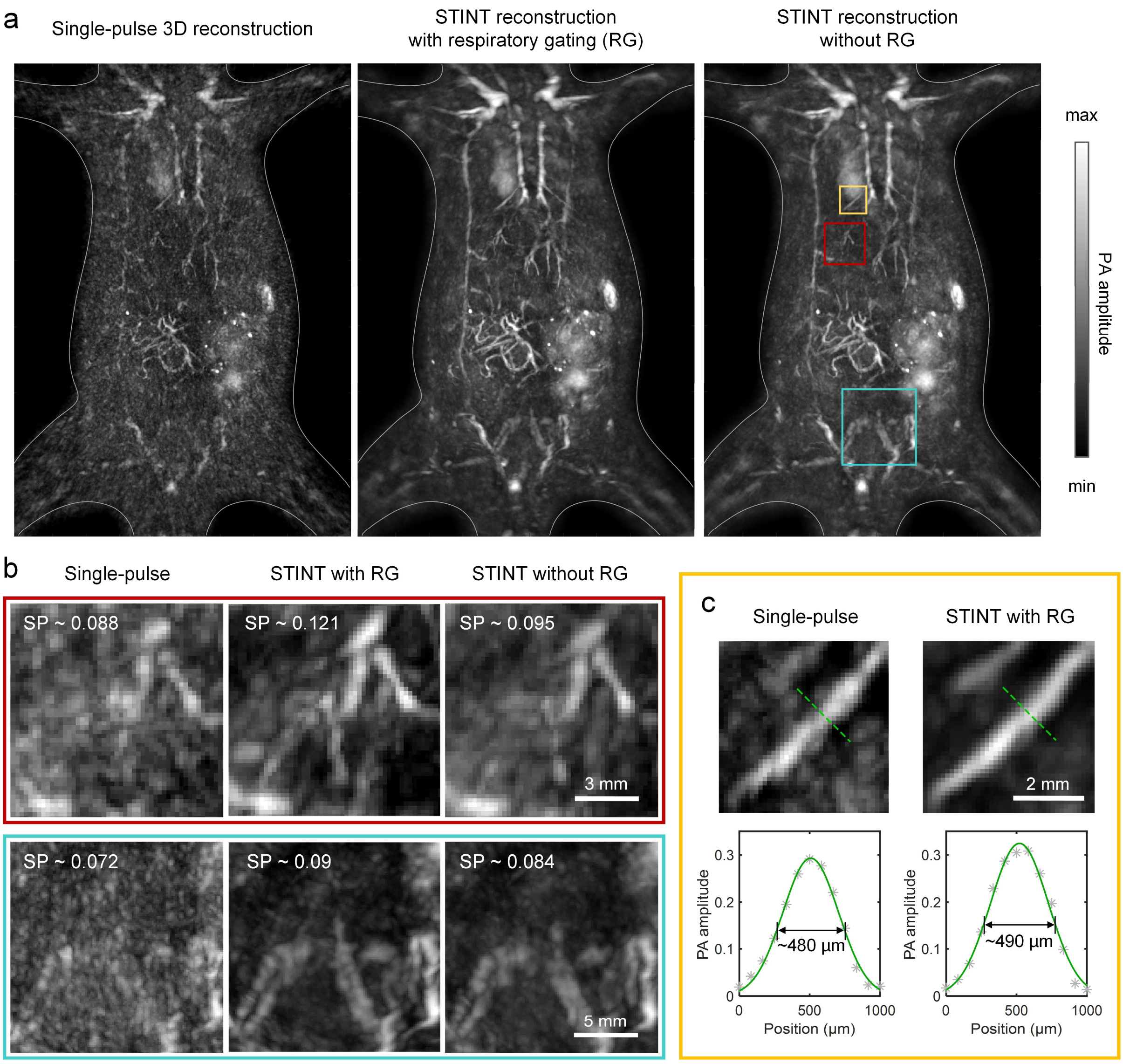

Figure S12. Quantification of respiratory motion effects in the STINT method.

a Comparison of whole-body imaging using single-pulse and STINT methods with and without respiratory gating. **b, c** Comparison of imaging details, with different regions corresponding to the boxes in **a**. RG, respiratory gating. SP, image sharpness.

Supplementary Tables

System name	Our 3D-PanoPACT	Caltech 3D-PACT[1, 2]	Canon PAI-04[3]	Zurich FONT[4]
**Array type**	f_c = 3.16 MHz<br>Spherical array with 1024 elements (2π solid angle)	f_c = 2.25 MHz<br>four arc-shaped arrays with 256×4 elements (2π solid angle)	f_c = 4 MHz<br>Spherical array with ~500 elements (2π solid angle)	f_c = 5 MHz<br>Spherical array with 512 elements ($\frac{7\pi}{9}$solid angle)
**Piezoelectric conversion mechanism**	Piezocomposite materials	Not demonstrated	CMUT	Piezocomposite materials
**Element Size**	5 mm in diameter (19.6 mm^2)	0.6 mm×0.7 mm (0.42 mm^2)	1.5 mm in diameter (1.77 mm^2)	1-2.5 mm in diameter for different versions (0.79-4.9 mm^2)
**Ratio of element size to the central wavelength (ROSW).**	10.53	1.05	4	3.33-8.33
**Array motion**	Small-angle rotation with optional translation	Large-angle rotation	Spiral translational scanning	Not demonstrated
**Geometric calibration**	Applied	Applied	Not demonstrated	Not demonstrated
**Image reconstruction**	Dual-speed-of-sound UBP algorithm	Dual-speed-of-sound UBP algorithm	Dual-speed-of-sound UBP algorithm	UBP algorithm
**Spatial resolution**	250 μm<br>(nearly isotropic)	370 μm<br>(nearly isotropic)	270 μm<br>(nearly isotropic)	150 μm<br>(nearly isotropic)

**In-vivo imaging depth**	~20 mm	10 mm in rat brains<br>15 mm in rat liver<br>40 mm in human breasts	~10 mm in human breasts	7 mm in mouse brain
**Imaging speed and well-resolved FOV**	1) 25 Hz single-wavelength imaging, FOV = 6 cm^3 (depending on the laser rate)<br>2) 10 Hz dual-wavelength whole-brain imaging, FOV = 3.2 cm^3 (depending on the laser rate)<br>3) 0.5 Hz brain base functional imaging, FOV = 3.2 cm^3<br>4) 10Hz whole-trunk imaging, FOV = 70.5 cm^3	1) 0.5 Hz functional imaging, FOV = 2.2 cm^3<br>2) 10 s for human breast imaging, FOV = 50.2 cm^3	2 minutes for human breast imaging, FOV = 46.2 cm^3	25 Hz for mouse brain functional imaging, FOV = 0.033 cm^3
**Acquisition time per voxel per laser pulse**	~11 ns	~ 4 µs	Not involved	~ 0.5 µs
**Functional imaging**	1) Hepatic artery mapping with a single wavelength<br>2) Hemodynamics during electrical stimulations on limbs<br>3) Hemodynamics during SNP administration in the Circle of Willis **(first proposed)**<br>4) Whole-trunk dynamics **(first proposed)**	Hemodynamics during<br>1) Hypoxic challenges<br>2) Deep to light anesthesia<br>3) Resting state<br>4) Electrical stimulations on limbs	S-factor<br>(i.e., estimated oxygen saturation)	1) Hemodynamics<br>2) GCaMP6 responses<br>during electrical stimulations on limbs

	5) High-spatiotemporal-resolution tracking of small molecule metabolic pathways at whole-body scale **(first proposed)**			
**Small animal whole-brain imaging**	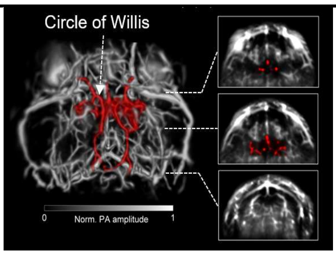<br>With a portion of the parietal bone removed (~ 8 mm in diameter) and the sutured scalp	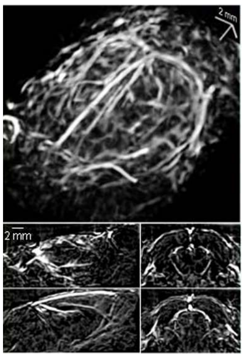<br>With thinned skull	Not demonstrated	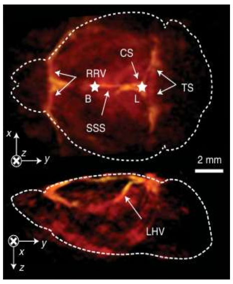<br>With intact scalp and skull
**Small animal whole-trunk imaging**	Structural imaging:<br>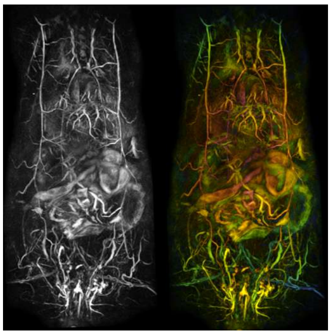<br>Functional imaging:<br>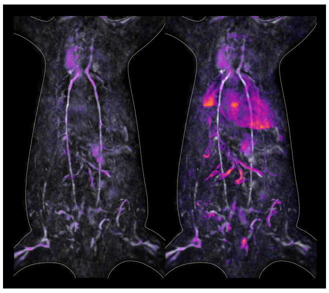	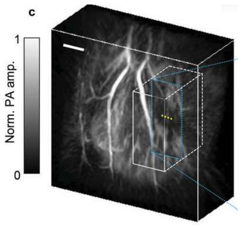	Not demonstrated	Not demonstrated

**Human breast imaging**	Not demonstrated	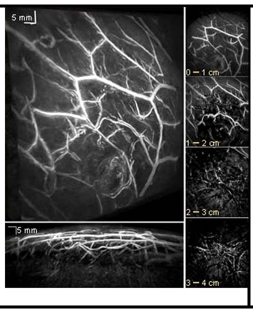	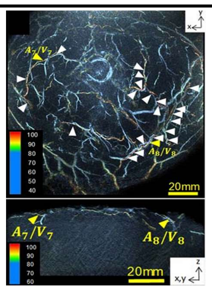	Not demonstrated

Table S1. Performance comparison of 3D-PanoPACT with other state-of-the-art three-dimensional PACT systems based on spherical array configuration. Note that in the comparison of the metric "Acquisition time per voxel per laser pulse" (row 6), the voxel size of different systems is calculated using a quarter of the central wavelength corresponding to the transducer array, for a fair comparison.

Imaging sites	Brain coronal images	Trunk cross-sections
**Previous 2D PACT results**	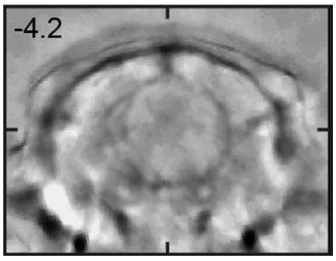<br>(from MSOT system[5])	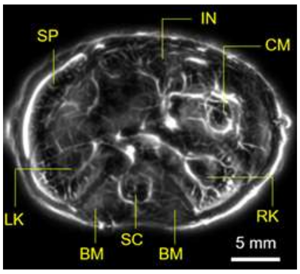<br>(from SIP-PACT system[6])
**Cross-sections from 3D images in 3D-PanoPACT**	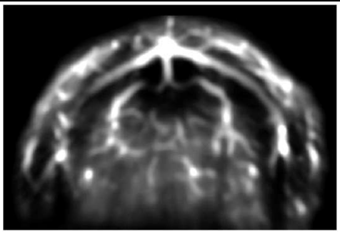	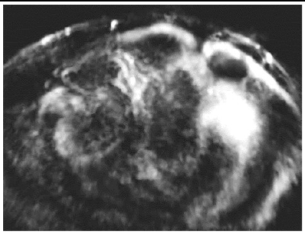

Table S2. Performance comparison of 3D-PanoPACT with other state-of-the-art 2D-PACT systems to demonstrate the imaging depth.

System name	Other PACT system[7]	Our 3D-PanoPACT
**Imaging results of whole-brain anatomy and the Circle of Willis**	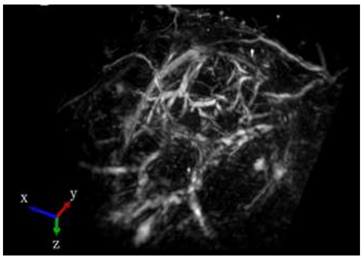	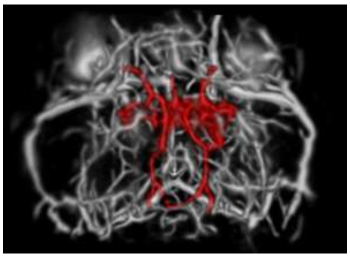
**Imaging time**	0.5 hour	2 s

Table S3. Comparison of 3D-PanoPACT and other PACT systems in imaging 3D whole-brain anatomy as well as the Circle of Willis.

System name	Our 3D-PanoPACT	Zurich SVOT[8]	fSVOT[9]	sSVOT[10]	Head-to-tail SVOT[11]
**Array type**	1024-element spherical array, r = 100 mm, coverage angle = 180°, f_c = 3.16 MHz	256-element spherical cap, r = 40 mm, coverage angle = 90°, f_c = 4 MHz	256-element spherical cap, r = 40 mm, coverage angle = 90°, f_c = 4 MHz	512-element spherical cap, r = 40 mm, coverage angle = 110°, f_c = 7 MHz	512-element spherical cap, r = 40 mm, coverage angle = 110°, f_c = 7 MHz
**Spatial resolution at center of FOV**	~200 µm	~200 µm	~200 µm	~130 µm	~150 µm
**Imaging speed and well-resolved FOV**	1) 25 Hz single-wavelength imaging, FOV = 6 cm^3 (depending on the laser rate)<br>2) 10 Hz dual-wavelength whole-brain imaging, FOV = 3.2 cm^3 (depending on the laser rate)<br>3) 0.5 Hz brain base functional imaging, FOV = 3.2 cm^3<br>4) 10 Hz whole-trunk imaging, FOV = 70.5 cm^3	1) 100 Hz single-wavelength imaging, FOV = 1 cm^3<br>2) whole-trunk imaging for 5 min	Mid-trunk imaging for 45 s	whole-trunk imaging for 1.8 s	whole-body imaging for 12 min

**Functional imaging**	1) Hepatic artery mapping with a single wavelength<br>2) Hemodynamics during electrical stimulations on limbs<br>3) Hemodynamics during SNP administration in the Circle of Willis **(first proposed)**<br>4) Whole-trunk dynamics **(first proposed)**<br>5) High-spatiotemporal-resolution tracking of small molecule metabolic pathways at whole-body scale **(first proposed)**	Organ-level tracing of ICG metabolism	Organ-level tracing of AuNP kinetics	Not demonstrated	Not demonstrated
**Real-time imaging**	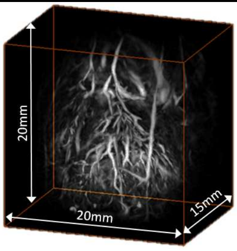<br>FOV = 6 cm^3	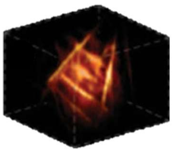<br>FOV = 1 cm^3	Not demonstrated	Not demonstrated	Not demonstrated

**Cross-sections**	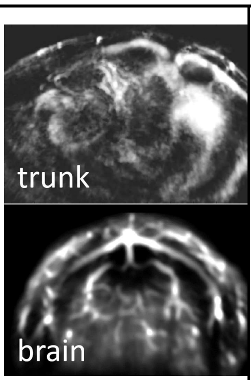	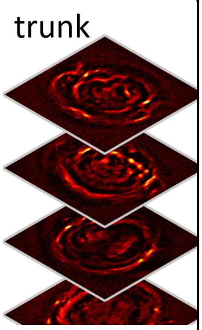	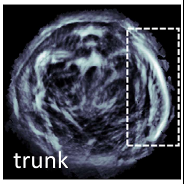	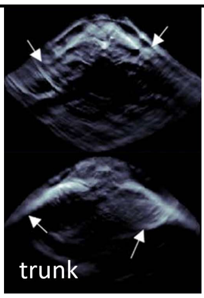 (acquired in 6.9 s)	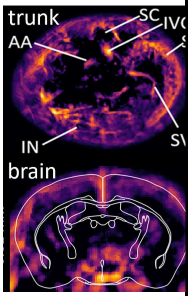
**3D Brain imaging**	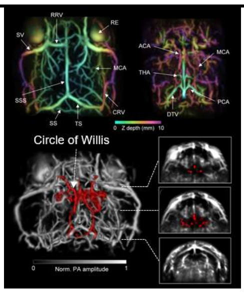	Not demonstrated	Not demonstrated	Not demonstrated	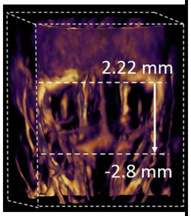
**3D Trunk imaging**	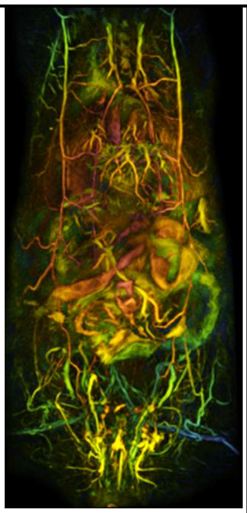	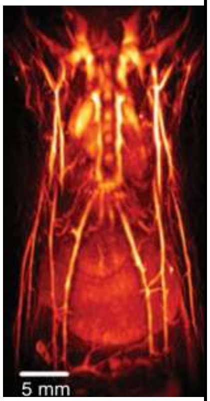	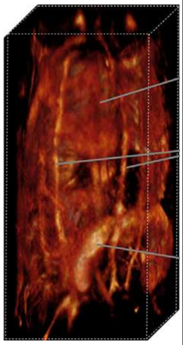	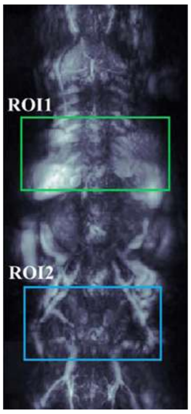	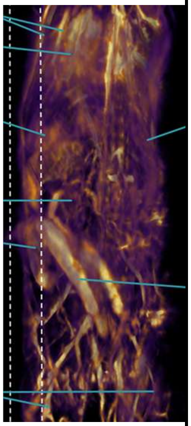

Table S4. Comparison of the state-of-the-art small-animal PACT systems with 3D-PanoPACT.

Supplementary Methods

Pseudo-Code and Description of the STINT method used in whole-body probe tracking (the first implementation form of the STINT method, Fig. 6b, c, Fig. 7e-g)

1. Respiratory gating

```
Initialize a 2D square matrix and set it to zero as the initial frame-to-frame cross-correlation matrix.
for ( nFramex = 1 to all frame numbers )
{
   for ( nFramey = 1 to all frame numbers )
   {
      Calculate the correlation coefficient matrix of the data segments of the nFramex and nFramey frames.
      Assign the off-diagonal value to the pixel at position ( nFramex, nFramey ) of the cross-correlation matrix.
   }
}
Calculate the mean of any dimension of the cross-correlation matrix and generate the phase curve.
Extract static frames using threshold segmentation.
```

2. Dual-Speed-of-Sound image reconstruction

```
Load raw data ← loadData().
Obtain an initial image estimate ← SingleSpeedReconstruction(data).
Determine the ellipsoidal parameters from the initial image ← AnalyzeImageForEllipsoid(image_initial).
Build ellipsoidal surface model ← GenerateEllipsoid(ellipsoid_params).
Calculate water sound speed based on temperature as the external sound speed ← CalculateWaterSoundSpeed(measured_temperature).
Optimize internal sound speed (Sound Speed looping Algorithm)
best_score ← -∞
best_speed_in ← undefined
for ( each speed in internal_speed_range )
{
      Reconstruct the image candidates ← DualSpeedReconstruction(data, ellipsoid_surface, sound_speed_out, speed).
```

```
    Calculate the quality score ← EvaluateImageQuality(image_candidate).
    if ( quality_score > best_score )
    {
        best_score ← quality_score.
        best_speed_in ← speed.
    }
    Reconstructed the final image ← DualSpeedReconstruction(data, ellipsoid_surface, sound_speed_out,
    best_speed_in).
}
```

3. The STINT method (the first implementation form)

```
Determine the rotational step angle between adjacent frames.
Set the reconstruction parameters.
Set the parameters of the ellipsoid surface.
Acquire static frames and their corresponding sequence numbers through respiratory gating.

Initialize all voxels in the to-be-reconstructed image as zero.
for ( nRot = the first to the last static frame number )
{
    Calculate the nRot-th angle based on the nRot number and the rotation step angle. The sign should be
    opposite to the mechanical direction of array rotation.
    Rotate the initial element positions for the nRot-th angle with the calibrated axis.
    Dual-Speed-of-Sound reconstruction with the rotated element positions.
    Add the reconstructed image to the initial voxels.
}
```

Supplymentary Reference

1. Lin, L. et al. High-speed three-dimensional photoacoustic computed tomography for preclinical research and clinical translation. *Nature communications* **12**, 882 (2021).
2. Tong, X. et al. Non-Invasive 3D Photoacoustic Tomography of Angiographic Anatomy and Hemodynamics of Fatty Livers in Rats. *Advanced Science* **10**, 2205759 (2023).
3. Matsumoto, Y. et al. Visualising peripheral arterioles and venules through high-resolution and large-area photoacoustic imaging. *Scientific reports* **8**, 14930 (2018).
4. Gottschalk, S. et al. Rapid volumetric optoacoustic imaging of neural dynamics across the mouse brain. *Nature biomedical engineering* **3**, 392-401 (2019).
5. Olefir, I. et al. Spatial and spectral mapping and decomposition of neural dynamics and organization of the mouse brain with multispectral optoacoustic tomography. *Cell Reports* **26**, 2833-2846. e2833 (2019).
6. Li, L. et al. Single-impulse panoramic photoacoustic computed tomography of small-animal whole-body dynamics at high spatiotemporal resolution. *Nature biomedical engineering* **1**, 0071 (2017).
7. Chen, Y. et al. Photoacoustic mouse brain imaging using an optical Fabry-Pérot interferometric ultrasound sensor. *Frontiers in Neuroscience* **15**, 672788 (2021).
8. Deán-Ben, X.L., Fehm, T.F., Ford, S.J., Gottschalk, S. & Razansky, D. Spiral volumetric optoacoustic tomography visualizes multi-scale dynamics in mice. *Light: Science & Applications* **6**, e16247-e16247 (2017).
9. Ron, A., Kalva, S.K., Periyasamy, V., Deán-Ben, X.L. & Razansky, D. Flash scanning volumetric optoacoustic tomography for high resolution whole-body tracking of nanoagent kinetics and biodistribution. *Laser & Photonics Reviews* **15**, 2000484 (2021).
10. Kalva, S.K., Dean-Ben, X.L. & Razansky, D. Single-sweep volumetric optoacoustic tomography of whole mice. *Photonics Research* **9**, 899-908 (2021).
11. Kalva, S.K., Deán-Ben, X.L., Reiss, M. & Razansky, D. Head-to-tail imaging of mice with spiral volumetric optoacoustic tomography. *Photoacoustics* **30**, 100480 (2023).
