# Supplementary material for: Cross-regional real-time visualization of systemic physiology and dynamics with 3D panoramic photoacoustic computed tomography (3D-PanoPACT)
Source: Nat Commun. 2025 Nov 18;16:10077. doi: 10.1038/s41467-025-65054-x (PMC12627095; doi:10.1038/s41467-025-65054-x)
Supplement: Supplementary file 2 — Description of Additional Supplementary Files [file 41467_2025_65054_MOESM2_ESM.pdf]

Description of Additional Supplementary Files

Supplementary Movie 1:

Real-time 3D imaging of the liver at a frame rate of 25 Hz (limited by the laser repetition rate). The results are shown in different views.

Supplementary Movie 2:

Real-time monitoring of the functional hemodynamics in the whole brain and the Circle of Willis in response to SNP administration at a frame rate of 0.5Hz.

Supplementary Movie 3:

Real-time monitoring of the cerebral hemodynamics introduced by electrical stimulation on forelimbs at a frame rate of 10 Hz. The red arrow represents the X-axis direction (the same below).

Supplementary Movie 4:

3D display of the anatomy of the whole trunk (in frontal and backside view) and the whole brain.

Supplementary Movie 5:

3D display and depth-encoded images of whole-trunk 3D dynamics (in frontal and backside view) at a frame rate of 10 Hz.

Supplementary Movie 6:

Dynamics of high-spatiotemporalresolution tracking of small molecule A1094 metabolic pathways at wholebody scale.
